# Suspected clopidogrel-associated hepatitis in a cat

**DOI:** 10.1177/20551169241278408

**Published:** 2024-11-06

**Authors:** Astrid L Kamp, Washma Yousofzai, Hans S Kooistra, Giorgia Santarelli, Ingeborg M van Geijlswijk

**Affiliations:** 1 Department Clinical Sciences, Division Internal Medicine of Companion Animals, Utrecht University, Utrecht, The Netherlands; 2 Department Population Health Sciences, IRAS Veterinary and Comparative Pharmacology Group (One Health Pharma) – Pharmacy, Utrecht University, Utrecht, The Netherlands

**Keywords:** Hypertrophic cardiomyopathy, clopidogrel, acute hepatitis, liver damage, adverse effect

## Abstract

**Case summary:**

Acute hepatitis and liver damage are rare adverse effects of clopidogrel in humans. In veterinary medicine, clopidogrel is mainly prescribed in the treatment of feline patients with cardiomyopathies. Little is known regarding the safety and adverse effects of clopidogrel in this group of patients. The limited number of studies scarcely report adverse effects. In this case report, a 6-year-old male castrated crossbred cat with the hypertrophic cardiomyopathy phenotype had signs of acute hepatitis after 5 weeks of clopidogrel treatment.

**Relevance and novel information:**

Evaluation of the case and review of the literature indicate that acute hepatitis might be a potential adverse effect of clopidogrel in feline patients. Therefore, hepatotoxicity should be taken into consideration when a feline patient shows clinical deterioration after the use of clopidogrel.

## Introduction

Cardiovascular disease is among the 10 most common causes of death in cats, with hypertrophic cardiomyopathy (HCM) being the most commonly diagnosed cardiopathy.^1–3^ HCM cats with left atrial enlargement have an increased risk of developing arterial thromboembolisms (ATEs).^4^ To reduce the risk of ATE and its devastating consequences, anti-thrombotic treatment is strongly advised in cats with an HCM phenotype on echocardiography, starting from stage B2 according to the American College of Veterinary Internal Medicine (ACVIM) classification. The current recommendation is the use of clopidogrel, an irreversible (ADP)2Y12 receptor blocker, in cats at risk of ATE, either alone or in combination with aspirin and/or rivaroxaban.^3,5^

Acute hepatitis and liver damage are rare adverse effects of clopidogrel in humans;^6,7^ however, in feline patients, little is known regarding the safety and adverse effects of clopidogrel. The limited number of studies scarcely report adverse effects.^8,9^ Because of the low number of cases included, these studies are generally unable to discover rare adverse effects of a drug. To give insight into rare adverse effects, postmarketing reports on patients are usually needed.

The aim of this case report was to share and evaluate experiences with the use of clopidogrel in a feline patient with HCM phenotype (ACVIM stage B2) that developed suspected acute hepatitis as a potential adverse effect.

## Case summary

A 6-year-old male castrated crossbred cat was presented with clinical signs of hyporexia/anorexia, hypersalivation, lethargy and hypodipsia. The clinical signs were present for 7 days and started 5 weeks after the start of clopidogrel treatment. Because of the anorexia, the owners were unable to administer the clopidogrel in the 5 days before presentation.

The cat was previously diagnosed at our hospital with congenital hypothyroidism (in March 2017) and treated with levothyroxine (50 µg PO q12h). An obstructive HCM phenotype was observed on echocardiography in April 2020 and treatment with atenolol (6.25 mg PO q24h) was instituted. A routine cardiology checkup in September 2022 revealed that the cardiomyopathy had progressed and showed left atrial enlargement. Treatment with clopidogrel (18.75 mg PO q24h) was initiated.

On presentation, physical examination disclosed mild tachypnea, mild dehydration and hypofrequent borborygmi. Initial laboratory investigation, including a complete blood count (CBC) and serum biochemistry profile, revealed markedly increased alanine aminotransferase (ALT) activity and bile acids, and mildly increased alkaline phosphatase (ALP) activity and total bilirubin. CBC was unremarkable, albumin and total proteins were mildly elevated, creatinine, gamma-glutamyl transferase (GGT), sodium, potassium and 1,2-*O*-dilauryl-*rac*-glycero-3-glutaric acid-(6'-methylresorufin) ester (DGGR) lipase, circulating calcium, electrolytes and phosphate were within their respective reference intervals (RIs) (see Table 1).

**Table 1:** Blood measurements before, during and after hospitalisation

Measurement (reference)	2017*	2018	2019	2020	2021	2022^†^	Day 1^‡^	Day 2	Day 3	Day 6^§^	Day 8^¶^	Day 13^∞^	Day 54^#^	8 months^#^	14 months^#^
ALT (39–95 U/l)	–	–	–	–	–	–	1131	–	859	781	1384	549	45	49	59
ALP (15–63 U/l)	–	–	–	–	–	–	73	–	–	–	83	48	21	17	16
Bile acids (<13 µmol/l)	–	–	–	–	–	–	40	–	4	10	–	–	2	1	<1
GGT (<8 U/l)	–	–	–	–	–	–	8	–	–	–	–	–	–	–	–
T_4_ (15–45 nmol/l)	<2	36	39	35	39	23	–	35	–	76	–	–	25	22	24
TSH (<0.60 µg/l)**	4.89	0.06	>0.03	0.09	1.75	0.97	–	0.06	–	–	–	–	–	0.03	0.1

An overview of all relevant clinical-chemistry parameters and over time. The yearly checkups before the consultation showed stable hypothyroidism, controlled with levothyroxine. In 2022, clopidogrel was started as a result of changes on echocardiogram. The cat was hospitalised for 8 days (days 1–8). On day 4, the cat clinically improved and started eating; on day 5, clopidogrel was restarted. On day 7, the cat stopped eating again. Because of ALT re-increasing after restarting clopidogrel, clopidogrel-induced hepatitis was suspected and clopidogrel was stopped.

* The cat was diagnosed with congenital hypothyroidism

† Clopidogrel was started as a result of changes on echocardiogram

‡ Five weeks after the start of clopidogrel, the cat was presented to our clinic

§ Clinical deterioration started

¶ Stopped clopidogrel owing to rising liver values

∞ First control visit, was clinically stable and eating well

# Control visit, no clinical signs were present

** There is no validated reference value for TSH in cats. The canine reference interval was used (<0.6 µg/l)

ALP = alkaline phosphatase; ALT = alanine aminotransferase; GGT = gamma-glutamyl transferase; TSH = thyroid-stimulating hormone

The next day, the cat was admitted for further work-up and stabilisation. Additional serum biochemistry, urine analysis and abdominal ultrasonography were performed. Urine analysis (taken via cystocentesis) showed a urine specific gravity of 1.012 g/ml and some traces of blood but was otherwise unremarkable. The bacterial culture of urine was negative. Abdominal ultrasonography identified liver, gallbladder, spleen, adrenal glands, pancreas, gastrointestinal tract, urinary bladder and abdominal lymph nodes within normal limits. The left kidney was slightly smaller than the right kidney but had normal corticomedullary distinction. Cytological examination of the liver conducted with fine-needle aspiration identified normal-looking hepatocytes with no signs of lipidosis. Additional serum biochemistry showed a total thyroxine concentration (T_4_) and a thyroid-stimulating hormone (TSH) concentration within their respective RIs.

The cat was hospitalised for a total of 7 days and received intravenous fluids (Sterofundin BG as maintenance and Sterofundin ISO as correction), antiemetics (metoclopramide initially on continuous rate infusion 1 mg/kg/day and later 0.2 mg/kg PO q6h, and ondansetron 0.3 mg/kg PO q12h). A nasal feeding tube was placed and enteral feeding started. Treatment with atenolol and levothyroxine was continued.

**Figure 1:**
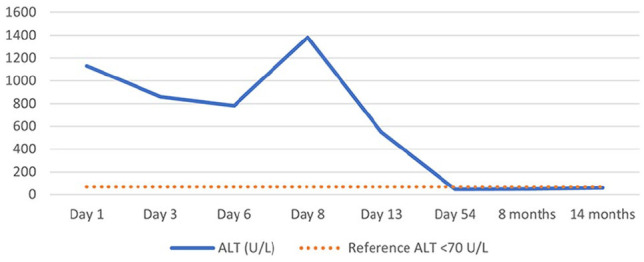
Alanine aminotransferase (ALT) progression over time. On day 1, the cat was presented at our clinic because of clinical deterioration for 7 days. The owners were unable to administer clopidogrel 5 days before this appointment. This was 5 weeks after clopidogrel treatment was started. On day 3, clopidogrel treatment was readministered. Because of clinical deterioration and rising ALT values, this was discontinued again on day 8. At the checkup on day 13, the ALT had dropped again; at the second checkup on day 54 after the original hospitalisation, all liver enzyme values were within their respective reference intervals. Eight months after the original hospitalisation, the cat came in for a routine re-check, had no clinical signs and all values were within their respective reference intervals

The cat clinically improved on the second day of hospitalisation and started to eat voluntarily. Clopidogrel was restarted and continued at the earlier dose. Initially, serum biochemistry showed a decline in ALT activity and bile acids dropped within the RI but began to rise again several days after clopidogrel treatment was restarted (Figure 1). On the same day, the T_4_ concentration increased. Clopidogrel was discontinued after recurrent elevation of liver values.

As a result of the clinical improvement and to avoid further stress, the owners decided to take the cat home, despite rising liver values, after 8 days of hospitalisation. Over the next 2 days at home, the cat began to deteriorate clinically (hyporexia, lethargy, vomiting once); however, the owners decided not to have the cat re-hospitalised but tried to forcefeed it at home.

Three days after the cat had returned home, the cat clinically improved again and came back for checkup 5 days after discharge (day 13). Biochemistry showed that ALT had dropped and ALP decreased to within its RI. The cat was clinically stable and eating. Two weeks after the last checkup, the cat was started on aspirin (20 mg three times per week). The cat returned for a cardiology and general checkup 1.5 months after discharge. By this time, the ALT, ALP, bile acids and T_4_ were all within their respective RIs. The cat remained clinically stable and was eating well.

At the routine cardiology checkup 8 months after hospitalisation, the cat remained in HCM phenotype ACVIM stage B2. Liver enzymes and bile acids were rechecked and were all within their respective RIs. The cat was doing well and did not show any clinical abnormalities in the months leading up to this appointment.

## Discussion

The initial decrease in ALT and bile acids in combination with the clinical improvement several days after the discontinuation of clopidogrel, and the subsequent increase of ALT combined with clinical deterioration after readministering clopidogrel, raised suspicion of clopidogrel-induced hepatotoxicity as a potential rare side effect of clopidogrel.

To determine whether a symptom or clinical sign is a potential adverse effect, several algorithms/models have been developed. The French imputability causality assessment scale is a model that assesses intrinsic accountability, that is, causality, by determining the pharmacological plausibility. This includes the dechallenge and rechallenge of the incriminated drug, evaluation of the effects of co-medication, patient-bound factors (eg, indication, comorbidities) and the way the drug is metabolised. For the extrinsic accountability, product information, literature and information from databases are used.^10^

The cat in this case report, diagnosed with congenital hypothyroidism and HCM, experienced hyporexia/anorexia, hypersalivation, lethargy and hypodipsia and had increased ALT activity 5 weeks after initiation of clopidogrel treatment. Problems with adherence already forced the owners to stop clopidogrel treatment for 5 days. After admittance, correction of anorexia and decreasing ALT, clopidogrel treatment was restarted. ALT increased to values higher than at admittance within 3 days after this rechallenge.

There was concurrent treatment with atenolol (cardiac disease) and levothyroxine (hypothyroidism) when the signs occurred. No clinical adverse effects have been mentioned or noticed for either drug during the prior usage, nor has either drug been reported to have an interaction with clopidogrel.^11^ This has been extensively investigated in humans, where clopidogrel has been used since 1997,^11–13^ but can also be concluded for cats based on how atenolol and levothyroxine are metabolised. Atenolol is eliminated from the body mostly (80–100%) unchanged through the kidneys.^14^ Levothyroxine (as an endogenous hormone) is metabolised in different parts of the body through deiodinases,^15,16^ affecting neither the metabolisation pathways of clopidogrel^12,17^ nor the drug–drug interaction routes as described by Lee et al^11^ for clopidogrel.

Although a pharmacokinetic interaction is unlikely, a pharmacodynamic interaction is plausible. Hepatitis and acute liver insufficiency are rare adverse effects of clopidogrel in humans.^6,7^ Elevation of liver enzymes has been associated with clopidogrel in 1–3% of human patients,^13^ with 10 recorded cases of hepatitis after clopidogrel use in human literature.^6,7,18–23^ However, atenolol too has a 1–2% occurrence rate of elevated liver enzymes in humans.^24^ Theoretically, the elevated ALT values could therefore have been a combined effect of atenolol and clopidogrel, and not something caused by clopidogrel alone. However, during the dechallenge with continued atenolol, the liver enzymes decreased and stayed within their respective reference rates, making this theory unlikely.

Levothyroxine is not known to cause any adverse hepatic effects, but hyperthyroidism is often associated with elevated liver enzyme activities.^25–27^ However, since our patient was hypothyroidic and the T_4_ values for this patient have been within the RI since levothyroxine was initiated, this cause for elevated ALT can be ignored.

Looking at the patient-bound factors, the highly variable metabolisation of clopidogrel in cats could explain why this patient reacted so poorly to clopidogrel.^28^ A study of 19 cats showed a high interindividual variability in clopidogrel metabolism associated with sex and genetic polymorphisms in the *P450 2C* gene.^29^ According to Zhai et al,^30^ high activities of CYP2C19 and CYP2B9 are risk factors for hepatocellular toxicity of clopidogrel. Other studies have found that P450 3A4/5 plays a more important role in the metabolisation of clopidogrel to the active metabolite in humans than the 2C enzyme.^17^ The feline isoforms of CYP3A4/5 are CYP3A131/132; the 2C are CYP2C21 and CYP2C41.^31–33^

Higher levels of clopidogrel metabolites (active/inactive) might affect the response to this drug, potentially resulting in adverse effects that are not seen in the majority of patients, such as hepatotoxicity.^34^ Zahno et al^34^ concluded that high human CYP3A4 activity and low cellular glutathione stores may be risk factors for clopidogrel-associated hepatocellular toxicity. Cats have naturally low glutathione stores and an inability to glucuronidate substances with a phenolic structure (ie, clopidogrel), which could increase the risk for developing clopidogrel-associated hepatocellular toxicity;^2,35–37^ however, whether the feline isoforms of UGT are involved in the glucuronidation of clopidogrel is yet to be determined. It is also unknown whether genetic polymorphism or other patient-bound factors caused the elevated ALT levels in this patient, but it is a potential explanation.

## Conclusions

Although no definite conclusion can be drawn, the available literature and the changes in clinical signs indicate that hepatotoxicity could indeed be a rare adverse effect in cats treated with clopidogrel and should be taken into consideration when a patient shows clinical deterioration after use of this drug. More publications on the use, adverse effects and long-term effect of clopidogrel in cats are needed.
